# LncRNA-TUSC7/miR-224 affected chemotherapy resistance of esophageal squamous cell carcinoma by competitively regulating DESC1

**DOI:** 10.1186/s13046-018-0724-4

**Published:** 2018-03-12

**Authors:** Zhi-wei Chang, Yong-xu Jia, Wei-jie Zhang, Li-jie Song, Ming Gao, Ming-jun Li, Rui-hua Zhao, Jing Li, Ya-li Zhong, Qiao-zhi Sun, Yan-ru Qin

**Affiliations:** Department of Oncology, The First Affiliated Hospital of Zhengzhou University, No.1 Jianshe East Road, Zhengzhou, 450052 Henan People’s Republic of China

**Keywords:** TUSC7, miR-224, DESC1, Chemotherapy resistance, Esophageal squamous cell carcinoma

## Abstract

**Background:**

This study aims to clarify the underlying mechanism for the tumor suppressive function of lnc TUSC7 in chemotherapy resistance of esophageal squamous cell carcinoma (ESCC).

**Methods:**

TUSC7, miR-224 and DESC1 expressions in ESCC tissues and cells were detected by qRT-PCR. Protein level of DESC1, EGFR and p-AKT were observed by Western blot. Overall survival was calculated using the Kaplan-Meier method. Dual-luciferase reporter gene assay and RIP assay were used to comfirm TUSC7 binding to miR-224, and miR-224 binding to DESC1. Cell proliferation, apoptosis, and colony formation was detected by MTT, Flow Cytometry and Colony formation assays.

**Results:**

TUSC7 was downregulated in ESCC tissues and cells, and low TUSC7 indicated worse overall survival. The analysis of bioinformatics softwares showed that TUSC7 specifically bound to miR-224, and we proved miR-224 was upregulated in ESCC and negatively correlated with TUSC7 expression. Overexpression of TUSC7/inhibition of miR-224 suppressed cell proliferation, colony formation and chemotherapy resistance of ESCC cells, and promoted cell apoptosis. In addition, we confirmed that miR-224 specifically bound to DESC1, and negatively correlated with DESC1. TUSC7 suppressed the proliferation and chemotherapy resistance of ESCC cells by increasing DESC1 expression via inhibiting miR-224. We also confirmed DESC1 inhibited chemotherapy resistance of ESCC cells via EGFR/AKT. Finally, in vivo experiments demonstrated that overexpression of TUSC7 decreased tumor growth and chemotherapy resistance.

**Conclusion:**

These findings suggested TUSC7 suppressed chemotherapy resistance of ESCC by downregulating miR-224 to modulate DESC1/EGFR/AKT pathway.

## Background

Esophageal cancer (EC) is the sixth most deadly cancer worldwide [1], which is caused by many factors, such as smoking, alcohol, lack of fruits and vegetables, and esophageal squamous cell carcinoma (ESCC) accounts for about 88% in EC [2]. Chemotherapy is an important clinical treatment of ESCC, and has gained certain therapeutic effects and less toxicity [3, 4]. Although the combined chemotherapy has been used for treating ESCC, acquired drug resistance remains a major clinical obstacle to achieve successful treatment [5–7], and the underlying mechanism of drug resistance in ESCC is still not fully revealed.

Differentially expressed in squamous cell carcinoma 1 (DESC1) belongs to the type II transmembrane serine protease (TTSP) family, which is an epithelial-specific enzyme that has been firstly identified by gene-expression analysis and found downregulated in squamous cell carcinoma of the head and neck region [8, 9]. Later, Zinovyeva et al. reported the expression of DESC1 was downregulated in tumor esophageal tissue [10]. Recently, Ng et al. found that DESC1 could act as a tumor suppressor and sensitized cells to apoptosis through downregulating EGFR/AKT pathway in ESCC [11]. However, the upstream moleculars that regulated DESC1 was still not clear.

microRNAs are small noncoding RNAs that may deeply involved in the development, progression and metastasis of cancer [12]. Numerous reports have been found that miRNAs were abnormally expressed in ESCC, such as miR-27, miR-652-5p, miR-21-5p, miR-107, etc. [13–15]. Reserachers have reported that miR-224 was overexpressed in ESCC tissues, and promoted proliferation and suppressed apoptosis of ESCC cells [16]. In addition, bioinformatics software [17] predicted there was potential binding site between miR-224 and 3’UTR of DESC1, predicting that DESC1 may be a direct target of miR-224. Thus, we studied miR-224 as a potential upstream molecular of DESC1.

Long non-coding RNA (lncRNA) are emerging as vital regulators that mediate cell cycle, autophagy and apoptosis, and act as oncogenes or tumor suppressor genes [18, 19]. It has been reported that lnc tumor suppressor candidate 7 (TUSC7) was downregulated and acted as a tumor suppressor in many cancers, such as colorectal cancer [20], glioma [21] and gastric cancer [22]. Therefore, we assume TUSC7 may also abnormally express in ESCC and participate in the progress of ESCC. Besides, bioinformatics software predicted there were potential binding sites between TUSC7 and miR-224. Hence, we predict that lnc TUSC7/miR-224 affect chemotherapy resistance of ESCC by regulating DESC1/EGFR/AKT pathway.

In this study, we demonstrated that TUSC7 was downregulated in ESCC, and overexpression of TUSC7/inhibition of miR-224 repressed proliferation of ESCC cells, promoted cell apoptosis, and inhibited chemotherapy resistance via DESC1. Low TUSC7 also decreased overall survival of patients with EC, and overexpression of TUSC7 inhibited colony formation in vitro and tumor volume and weight in vivo. Our study proved that TUSC7 affected chemotherapy resistance of ESCC and clarified the molecular mechanism underlying this function.

## Methods

### Patients, samples and cell culture

This study was approved by Ethics Committee of Zhengzhou University, and informed consent was obtained from each patient. A total of 62 EC patients with primary ESCC who took Neoadjuvant chemotherapy after esophagectomy in The First Affiliated Hospital of Zhengzhou University were recruited in this study. ESCC tissues and their paired adjacent normal esophageal epithelial tissues were collected and stored at − 80 °C. According to the National Comprehensive Cancer Network esophageal cancer guideline, the normal tissues were at least 5 cm away from the primary lesion.

Combined chemotherapy for the treatment of ESCC was cisplatin, 5-Fu and adriamycin, or cisplatin, 5-Fu and paclitaxel. According to Response Evaluation Criteria in Solid Tumors (RECIST) guideline, patients were divided into complete response (CR), partial response (PR), stable disease (SD), and progressive disease (PD) after complete chemotherapy. Patients with CR and PR were defned as responders, whereas those with SD and PD were defned as non-responders.

ESCC cell lines (TE-13, KYSE140, EC9706, KYSE30) and human esophageal epithelial cells (Het-1A) were purchased from Cell Bank of TypeCulture Collection of Chinese Academy of Sciences (Shanghai, China), and cultured in RPMI1640 supplemented with 10% fetal bovine serum (Gibco, USA) in 5% CO_2_ incubator under 37 °C.

### Cell transfection

Cells were seeded into a culture plate and grown to 70-80% confluence for cell transfection. si-TUSC7-1, si-TUSC7-2, pcDNA-TUSC7, miR-224 mimic, miR-224 inhibitor, si-DESC1, LV-TUSC7 and their non-specific control were synthesised by Invitrogen (Shanghai, China), and were transfected into cells using Lipofectamine 2000 (Invitrogen, USA).

### Quantitative real-time PCR (qRT-PCR)

Total RNA from ESCC tissues, paired adjacent normal esophageal epithelial tissues, ESCC cell lines and Het-1A cells were isolated by TRIzol Reagent (Invitrogen, USA) according to manufacturer’s instructions. The expressions of TUSC7, miR-224 and DESC1 were neasured by PowerUp™ SYBR™ Green Master Mix (Applied Biosystems, USA), and qRT-PCR was performed by QuantStudio® 3 RCR Real-Time PCR systems (Applied Biosystems, USA). The relative TUSC7, miR-224 and DESC1 expressions were determined by comparative method 2^-ΔΔCt^.

### Dual luciferase reporter assays

Luciferase report gene vectors (pRL-TK, Promega) containing TUSC7 Wild Type (WT) or TUSC7 mutant 1 (Mut 1) or TUSC7 mutant 2 (Mut 2) were transfected into HEK293T cells. Luciferase report gene vectors containing DESC1 3’UTR WT or DESC1 3’UTR Mut (400 ng) was transfected into HEK293T cells with 40 ng pRL-TK vectors (Promega, USA). miR-224 mimic or miR-224 inhibitor or NC was co-transfected with reporter plasmids for 48 h. Cells were collected to measure luciferase activity by dual Glo™ Luciferase Assay System (Promega).

### MTT assay

Cell proliferation was evaluated by the 3-(4,5-dimethylthiazol-2-yl)-2,5-diphenyl-tetrazolium bromide (MTT) assay. 8 × 10^3^ EC9706 or KYSE30 cells were seeded in each well of a 96-well plate. After overnight incubation, absorbance value was read at a reference of 450 nm. The inhibition rate (%) = (1-OD value of treated group/OD value of the control group) × 100%.

### Colony formation assay

Colony formation assay was conducted as previously reported [23]. EC9706 or KYSE30 cell suspension (3500 cells) were uniformly dispersed, seeded onto six-well plates and grew over a span of 2 weeks in 5% CO_2_ incubator under 37 °C. Then, cells were fixed with 10% formalin (Sigma, USA) and stained with 1 × diluted Giemsa reagent (Sigma, USA).

### Flow cytometry

Apoptosis was detected by Annexin V-FITC Apoptosis Detection Kit (Beyotime, China). EC9706 or KYSE30 cells with different treatments were collected, centrifuged at 1500 rpm for 5 min, and resuspend with PBS. The supernatant was discarded, and 1 × Annexin V binding buffer (195 μL) was used to resuspend the cells. Annexin-V/FITC (5 μL) was added to the cell suspension. Then, PI (10 μL) was added to the cells and incubated at room temperature in the dark for 15 min. FACScalibur cytometer (Becton Dickinson, USA) was used to observe cell apoptosis.

### RNA immunoprecipitation (RIP) assays

Magna RIP™ RNA-Binding Protein Immunoprecipitation Kit (Millipore, USA) was used for RIP experiments according to the manufacturer’s instructions. Antibody for RIP assays of endogenous polycomb repressive complex2 (EZH2) was purchased from Invitrogen. AGO2 was assessed by IP-western, and TUSC7 level was detected by qRT-PCR.

### Cisplatin or 5-Fu treatment

Cisplatin (DDP) and 5-Fu were purchased from Sigma and dissolved in DMSO. EC9706 or KYSE30 cells transfected with pcDNA or pcDNA-TUSC7 were incubated with cisplatin (0, 1, 2, 4, 8, 16 μM) or 5-Fu (0, 1, 4, 16, 32, 64 μM) for 48 h.

### Establishment of acquired resistance cell lines

The drug-resistant cell lines were established by the increase of the cisplatin concentration. EC9706 or KYSE30 cells at the logarithmic phase were seeded into culture solution containing DDP with the low concentration started from 0.5 μM. 48 h later, the solution was discarded and fresh solution was added. After digestion, 1 μM DDP was added for the treatment for 48 h. With this procedure of changing solution and gradually increasing DDP concentration, cell lines that can resistent to 10 μM DDP were obtained, and named EC9706/DDP or KYSE30/DDP.

### Western blot analysis

Proteins from ESCC tissues, paired adjacent normal esophageal epithelial tissues, ESCC cell lines and Het-1A cells were extracted using RIPA buffer (Thermo Scientific, USA), and protein concentrations were detected by BCA Protein Assay kit (Pierce Biotechnology, USA). Protein samples (50 μg) were isolated in 10% SDS-polyacrylamide gel electrophoresis (SDS-PAGE), then transferred to polyvinylidene difluoride (PVDF) membranes (Invitrogen, USA) and blocked in 5% non-fat dried milk. Then, the membranes were probed with first primary antibody anti-DESC1 (1:1000, Signalway Antibody, USA), anti-EGFR (1:1000, Invitrogen, USA), anti-p-AKT (1:1000, Invitrogen, USA), anti-AKT (1:1000, Cell Signaling, USA) and anti-GAPDH (1:1000, Invitrogen, USA) and the secondary horseradish peroxidase-conjugated antibody (Invitrogen, USA). GAPDH was used as the internal loading control.

### Xenograft experiments

This study was approved by the Ethics Committee of Animal Experiments of Zhengzhou University. KYSE30 cells were transfected with LV-NC or LV-TUSC7. 1 × 10^6^ cells in 150 μl of culture medium were inoculated subcutaneously into 24 female nude mice at 5-weeks of age. Seven days later, mice were intraperitoneally injected with 2 mg/kg cisplatin or PBS every 5 days for 20 days. Therefore, the mice were divided into four groups with six mice in each group: LV-NC + PBS, LV-TUSC7 + PBS, LV-NC + cisplatin, and LV-TUSC7 + cisplatin groups. Tumor volumes and weight were recorded. Tumor volume = (length×width^2^)/2.

### Bioinformatic tools

LncBase v.2 was used for predicting the potential binding sites between TUSC7 and miR-224. LncBase v.2 provides users with functional information about the microRNAs and their interaction with lncRNAs in many species. MicroRNA.org resource was used for predicting the potential binding sites between miR-224 and 3’UTR of DESC1. MicroRNA.org provides users with functional information about the microRNAs and their interaction with target genes in many species.

### Statistical analysis

All experiments were performed in triplicate. SPSS 18.0 software was used for data analysis, and the result was expressed as mean ± standard deviation. The overall survival was calculated using the Kaplan-Meier method. One-way ANOVA and t test were used for the data analysis, with *P* < 0.05 considered statistically significant.

## Results

### Low expression of TUSC7 in ESCC tissues and cells

**Fig. 1:**
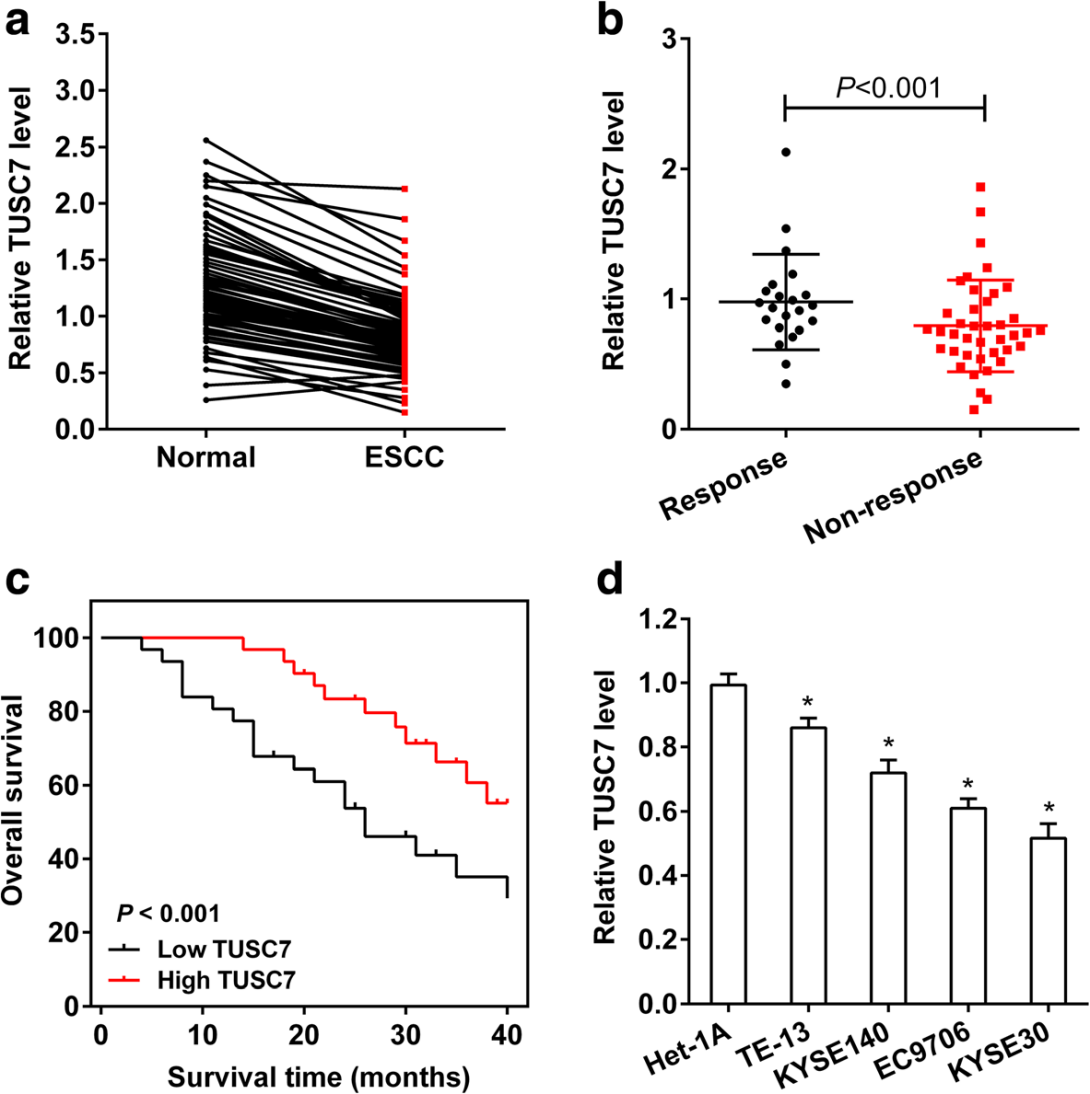
TUSC7 expression in ESCC tissues and cells. **a** Sixty-two ESCC tissues and adjacent normal esophageal epithelial tissues were collected. qRT-PCR showed that TUSC7 expression was downregulated in ESCC tissues. **b** Patients were divided into chemotherapy Response group (*n* = 22) and Non-response group (*n* = 40) according to the patient’s response to chemotherapy. qRT-PCR showed that TUSC7 expression was upregulated in chemotherapy Response group. **c** Kaplan-Meier survival analysis showed that patients with lower TUSC7 levels (*n* = 31) had poorer overall survival than patients with high TUSC7 levels (*n* = 31). **d** qRT-PCR was used to detect TUSC7 expression in ESCC cells (TE-13, KYSE140, EC9706, KYSE30) and human esophageal epithelial cells (Het-1A). Compared with Het-1A cells, TUSC7 expression was downregulated in ESCC cells with lowest expression in KYSE30 cells and highest in TE-13 cells

To determine whether TUSC7 was abnormally expressed in ESCC tissues and cells, qRT-PCR and Kaplan-Meier survival analysis were used to detect TUSC7 expression and overall survival of ESCC patients. We observed that TUSC7 level was downregulated in ESCC tissues than normal tissues (Fig. 1a), and upregulated in chemotherapy response group than chemotherapy non-response group (Fig. 1b). In addition, patients with lower TUSC7 levels had poorer overall survival (Fig. 1c), and there were significant associations between TUSC7 levels with tumor size and pathological staging (Table 1). Compared with Het-1A cells, TUSC7 expression was downregulated in ESCC cells with lowest expression in KYSE30 cells and highest in TE-13 cells (Fig. 1d).

**Table 1:** Association of TUSC7 expression with clinicopathological features from ESCC patients

Characteristics	Expression of TUSC7		*P*^a^ value
	Low(*n* = 31)	High(*n* = 31)	
Sex			0.408
Male	23	20	
Female	8	11	
Age			0.596
≤ 60	10	12	
> 60	21	19	
Tumor size (cm)			0.013*
≤ 5	17	26	
> 5	14	5	
Lymph node mestasis			0.127
Yes	19	13	
No	12	18	
Pathological Staging			0.022*
I + II	10	19	
III + IV	21	12	
Smoking status			0.430
Ever/current	10	13	
Never	21	18	
Alcohol consumption			0.543
Ever/current	6	8	
Never	25	23	

*P < 0.05

^a^Chi-square test

### TUSC7 regulated the expression of miR-224

**Fig. 2:**
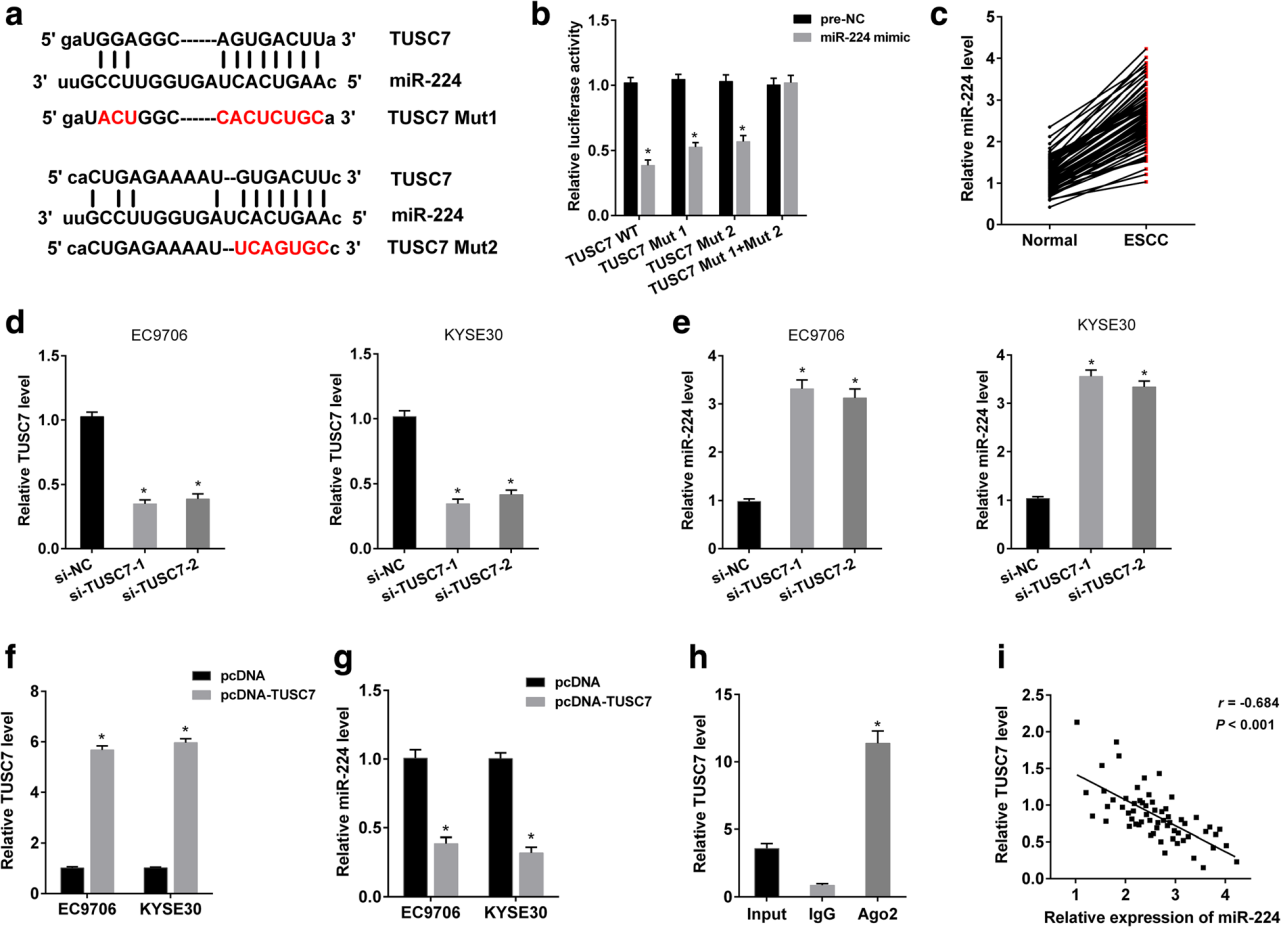
TUSC7 regulated the expression of miR-224. **a** Online bioinformatics software predicted there were two potential binding sites between TUSC7 and miR-224, and we mutated the two sites. **b** Luciferase reporter gene vector containing TUSC7 WT or TUSC7 mutant 1 or (and) TUSC7 mutant 2, and miR-224 or pre-NC were co-transfected into HEK293T cells. Dual-luciferase reporter gene assay showed that miR-224 mimic decreased the activity of TUSC7 WT, TUSC7 mutant 1 and TUSC7 mutant 2, and there was no significant difference in the activity in TUSC7 mutant 1 + mutant 2, suggesting miR-224 could bind to the two sites of TUSC7. **c** miR-224 expression was upregulated in ESCC tissue. **d-e** EC9706 or KYSE30 cells were transfected with si-NC or si-TUSC7-1 or si-TUSC7-2. qRT-PCR showed that si-TUSC7-1 or si-TUSC7-2 downregulated TUSC7 expression (**d**), and increased miR-224 expression (**e**). **f-g** EC9706 or KYSE30 cells were transfected with pcDNA or pcDNA-TUSC7. qRT-PCR showed that pcDNA-TUSC7 upregulated TUSC7 expression (**f**), and decreased miR-224 expression (**g**). H. KYSE30 cell lysate was treated with Ago2 antibody for RNA immunoprecipitation (RIP). qRT-PCR showed that TUSC7 was significantly increased in Ago2 than IgG. I. TUSC7 expression and miR-224 expression was negatively correlated in ESCC tissues

According to the prediction of online bioinformatics software, there were potential binding sites between TUSC7 and miR-224 (Fig. 2a). Dual-luciferase reporter gene assay showed that miR-224 mimic significantly decreased the activity of TUSC7 WT, TUSC7 mutant 1 and TUSC7 mutant 2, and there was no significant change in the activity in TUSC7 mutant 1 + mutant 2, suggesting miR-224 could bind to the two sites of TUSC7 (Fig. 2b). And we found miR-224 expression was upregulated in ESCC tissue (Fig. 2c). Besides, si-TUSC7-1 or si-TUSC7-2 downregulated TUSC7 expression (Fig. 2d), and increased miR-224 expression (Fig. 2e). pcDNA-TUSC7 upregulated TUSC7 expression (Fig. 2f), and decreased miR-224 expression (Fig. 2g). Moreover, KYSE30 cell lysate was treated with Ago2 antibody for RIP, and TUSC7 was significantly increased in Ago2 than IgG (Fig. 2h). Finally, there was a negative correlation between TUSC7 and miR-224 expressions in ESCC tissues (Fig. 2i). All these findings suggested that TUSC7 negatively regulated the expression of miR-224.

### Overexpression of TUSC7 or inhibition of miR-224 inhibited proliferation of ESCC cells and promoted cell apoptosis

**Fig. 3:**
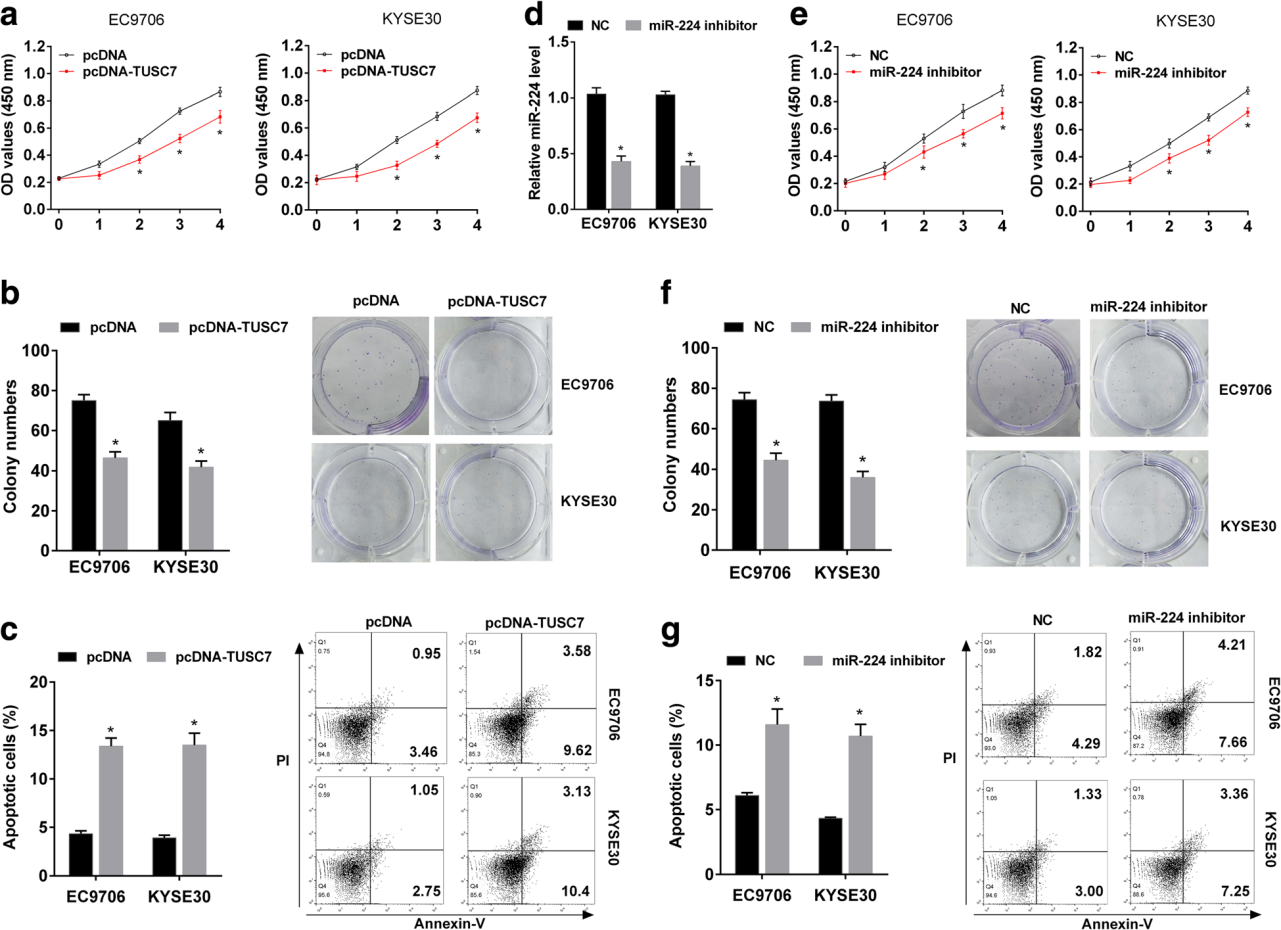
Overexpression of TUSC7 or inhibition of miR-224 inhibited proliferation of ESCC cells and promoted cell apoptosis. **a-c** EC9706 or KYSE30 cells were transfected with pcDNA or pcDNA-TUSC7. MTT assay showed that overexpression of TUSC7 inhibited proliferation of ESCC cells (**a**). Colony formation assay showed that overexpression of TUSC7 inhibited colony formation (**b**). Flow cytometry showed that overexpression of TUSC7 promoted apoptosis of ESCC cells (**c**). D-E. EC9706 or KYSE30 cells were transfected with NC or miR-224 inhibitor. qRT-PCR showed that miR-224 expression was downregulated (**d**). MTT assay showed that miR-224 inhibitor inhibited proliferation of ESCC cells (**e**). Colony formation assay showed that miR-224 inhibitor inhibited colony formation (**f**). Flow cytometry showed that miR-224 inhibitor promoted apoptosis of ESCC cells (**g**)

To figure out the effect of TUSC7 and miR-224 on the proliferation and apoptosis of ESCC cells, EC9706 or KYSE30 cells were transfected with pcDNA or pcDNA-TUSC7 or NC or miR-224 inhibitor. We found overexpression of TUSC7 inhibited proliferation of ESCC cells (Fig. 3a), inhibited colony formation (Fig. 3b), and promoted apoptosis of ESCC cells (Fig. 3c). miR-224 expression was downregulated after treated with miR-224 inhibitor (Fig. 3d). In addition, miR-224 inhibitor inhibited proliferation of ESCC cells (Fig. 3e), inhibited colony formation (Fig. 3f), and promoted apoptosis of ESCC cells (Fig. 3g).

### Overexpression of TUSC7 or inhibition of miR-224 inhibited chemotherapy resistance of ESCC cells

**Fig. 4:**
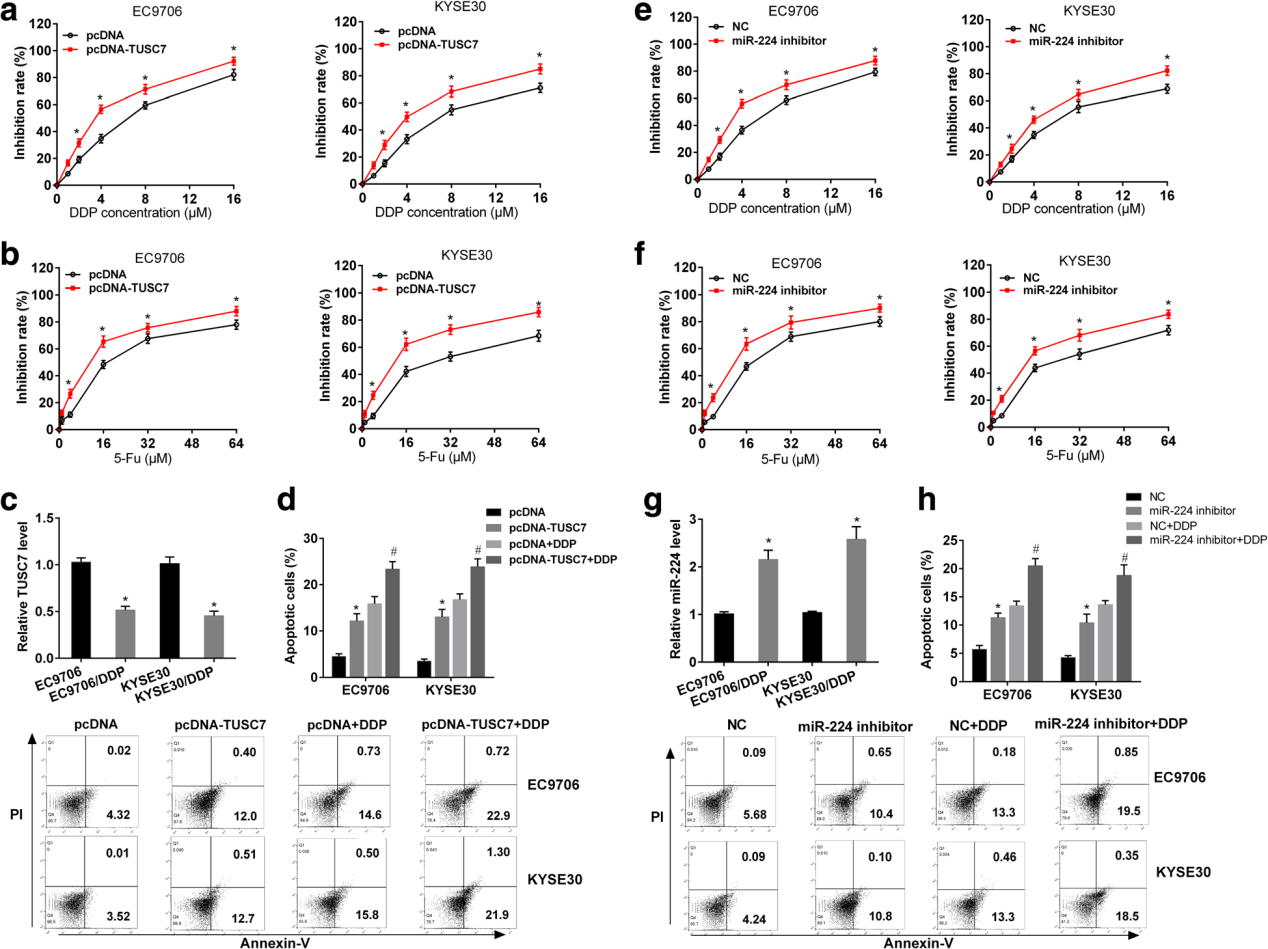
Overexpression of TUSC7 or inhibition of miR-224 inhibited chemotherapy resistance of ESCC cells. **a** EC9706 or KYSE30 cells transfected with pcDNA or pcDNA-TUSC7 were treated with cisplatin (0, 1, 2, 4, 8, 16 μM) for 48 h. Inhibition rate increased with the increase of concentration of cisplatin, and overexpression of TUSC7 increased the inhibition effect of cisplatin on ESCC cells, indicating overexpression of TUSC7 inhibited chemotherapy resistance of ESCC cells. **b** EC9706 or KYSE30 cells transfected with pcDNA or pcDNA-TUSC7 were treated with 5-Fu (0, 1, 4, 16, 32, 64 μM) for 48 h. Inhibition rate increased with the increase of concentration of 5-Fu, and overexpression of TUSC7 increased the inhibition effect of 5-Fu on ESCC cells. **c** TUSC7 level was detected in EC9706 and drug-resistant EC9706/DDP cells or KYSE30 and drug-resistant KYSE30/DDP cells. TUSC7 level was downregulated in drug-resistant ESCC cells. **d** EC9706 or KYSE30 cells transfected with pcDNA or pcDNA-TUSC7 were treated with 2 μM cisplatin for 48 h. Overexpression of TUSC7 promoted cisplatin induced apoptosis of ESCC cells. **e** EC9706 or KYSE30 cells transfected with NC or miR-224 inhibitor were treated with cisplatin (0, 1, 2, 4, 8, 16 μM) for 48 h. Inhibition rate increased with the increase of concentration of cisplatin, and miR-224 inhibitor increased the inhibition effect of cisplatin on ESCC cells, suggesting miR-224 inhibitor inhibited chemotherapy resistance of ESCC cells. **f** EC9706 or KYSE30 cells transfected with NC or miR-224 inhibitor were treated with 5-Fu (0, 1, 4, 16, 32, 64 μM) for 48 h. Inhibition rate increased with the increase of concentration of 5-Fu, and miR-224 inhibitor increased the inhibition effect of 5-Fu on ESCC cells. **g** miR-224 level was detected in EC9706 and drug-resistant EC9706/DDP cells or KYSE30 and drug-resistant KYSE30/DDP cells. miR-224 level was upregulated in drug-resistant ESCC cells. **h** EC9706 or KYSE30 cells transfected with NC or miR-224 inhibitor were treated with 2 μM cisplatin for 48 h. miR-224 inhibitor promoted cisplatin induced apoptosis of ESCC cells

To investigate the effect of TUSC7 and miR-224 on chemotherapy resistance of ESCC cells, EC9706 or KYSE30 cells transfected with pcDNA or pcDNA-TUSC7 were treated with cisplatin or 5-Fu. As shown in Fig. 4a and b, inhibition rate increased with the increase of concentration of cisplatin or 5-Fu, and overexpression of TUSC7 increased the inhibition effect of cisplatin or 5-Fu on ESCC cells, indicating overexpression of TUSC7 inhibited chemotherapy resistance of ESCC cells. We also detected TUSC7 level in EC9706 and drug-resistant EC9706/DDP cells or KYSE30 and drug-resistant KYSE30/DDP cells, and found TUSC7 level was downregulated in drug-resistant ESCC cells (Fig. 4c). And overexpression of TUSC7 promoted cisplatin-induced apoptosis of ESCC cells (Fig. 4d). According to Fig. 4e and f, inhibition rate increased with the increase of concentration of cisplatin or 5-Fu, and miR-224 inhibitor increased the inhibition effect of cisplatin or 5-Fu on ESCC cells, suggesting miR-224 inhibitor inhibited chemotherapy resistance of ESCC cells. We further detected miR-224 level in EC9706 and drug-resistant EC9706/DDP cells or KYSE30 and drug-resistant KYSE30/DDP cells, and observed miR-224 level was upregulated in drug-resistant ESCC cells (Fig. 4g). Also, miR-224 inhibitor promoted cisplatin induced apoptosis of ESCC cells (Fig. 4h).

### miR-224 targetedly regulated DESC1 expression

**Fig. 5:**
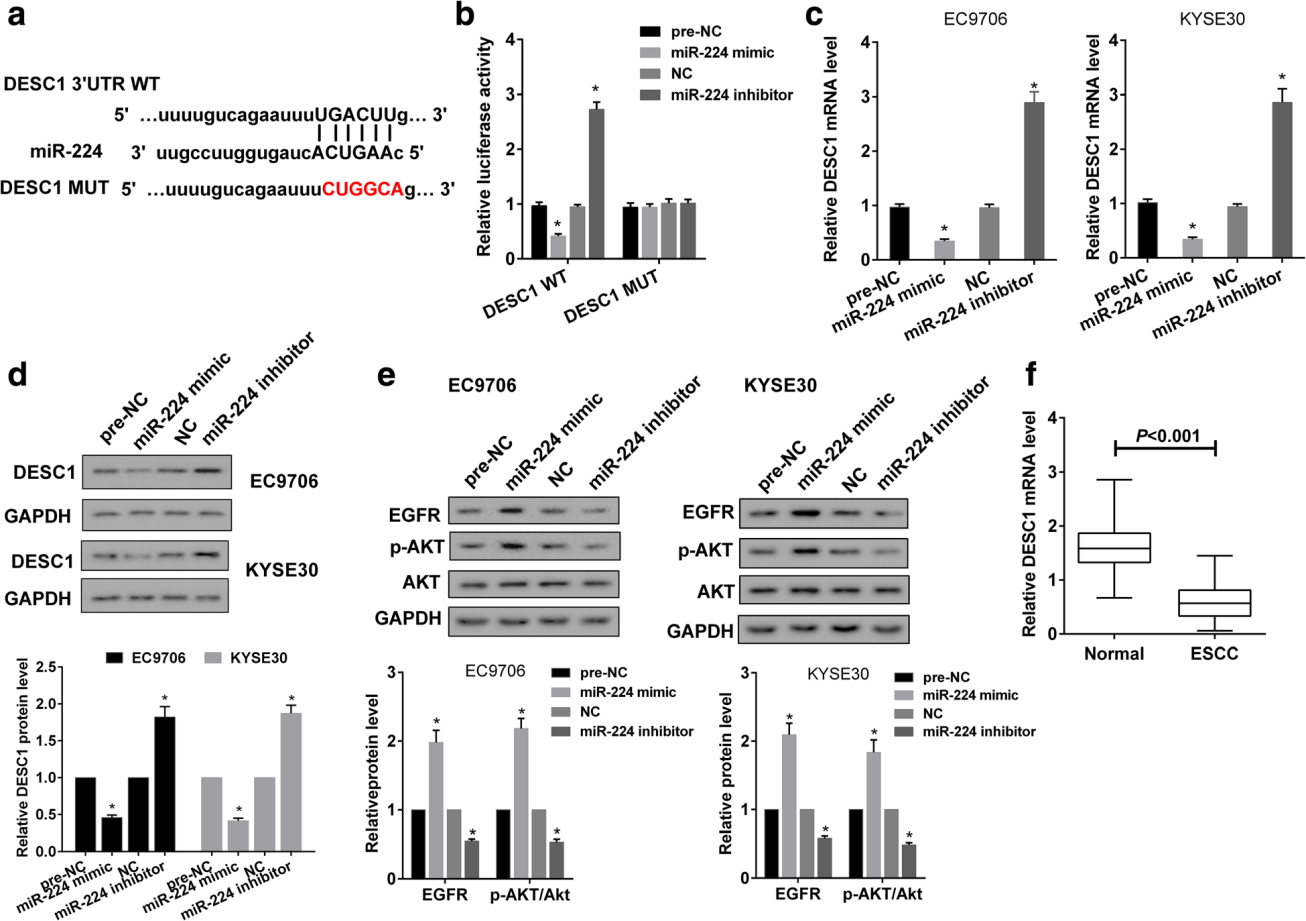
miR-224 targetedly regulated DESC1 expression. **a** Online bioinformatics software microrna.org predicted there was potential binding site between miR-224 and 3’UTR of DESC1. **b** Luciferase reporter gene vector containing DESC1 3’UTR WT or DESC1 3’UTR MUT, miR-224 mimic or pre-NC or miR-224 inhibitor or NC were co-transfected into HEK293T cells. Dual-luciferase reporter gene assay showed that miR-224 mimic or miR-224 inhibitor decreased or increased the activity of DESC1 WT, and did not significantly change the activity of DESC1 MUT. **c-e** EC9706 or KYSE30 cells were transfected with miR-224 mimic or miR-224 inhibitor or controls. qRT-PCR and Western blot showed that miR-224 mimic or miR-224 inhibitor significantly decreased or increased mRNA level (**c**) and protein level (**d**) of DESC1, and increased or decreased protein level of EGFR and p-AKT (**e**). **f** mRNA level of DESC1 was downregulated in ESCC tissues

Regulation of miR-224 on DESC1 was confirmed by luciferase reporter gene in HEK293T cells. Online bioinformatics software microrna.org predicted there was potential binding site between miR-224 and 3’UTR of DESC1 (Fig. 5a). Dual-luciferase reporter gene assay showed that miR-224 mimic or miR-224 inhibitor significantly decreased or increased the activity of DESC1 WT, and there was no significant difference in the activity of DESC1 MUT (Fig. 5b). Moreover, miR-224 mimic or miR-224 inhibitor significantly decreased or increased mRNA level (Fig. 5c) and protein level (Fig. 5d) of DESC1, and increased or decreased protein levels of EGFR and p-AKT (Fig. 5e). We also found mRNA level of DESC1 was downregulated in ESCC tissues (Fig. 5f). These findings proved that DESC1 was a direct target of miR-224.

### TUSC7 inhibited cell proliferation and chemotherapy resistance via miR-224-regulated DESC1

**Fig. 6:**
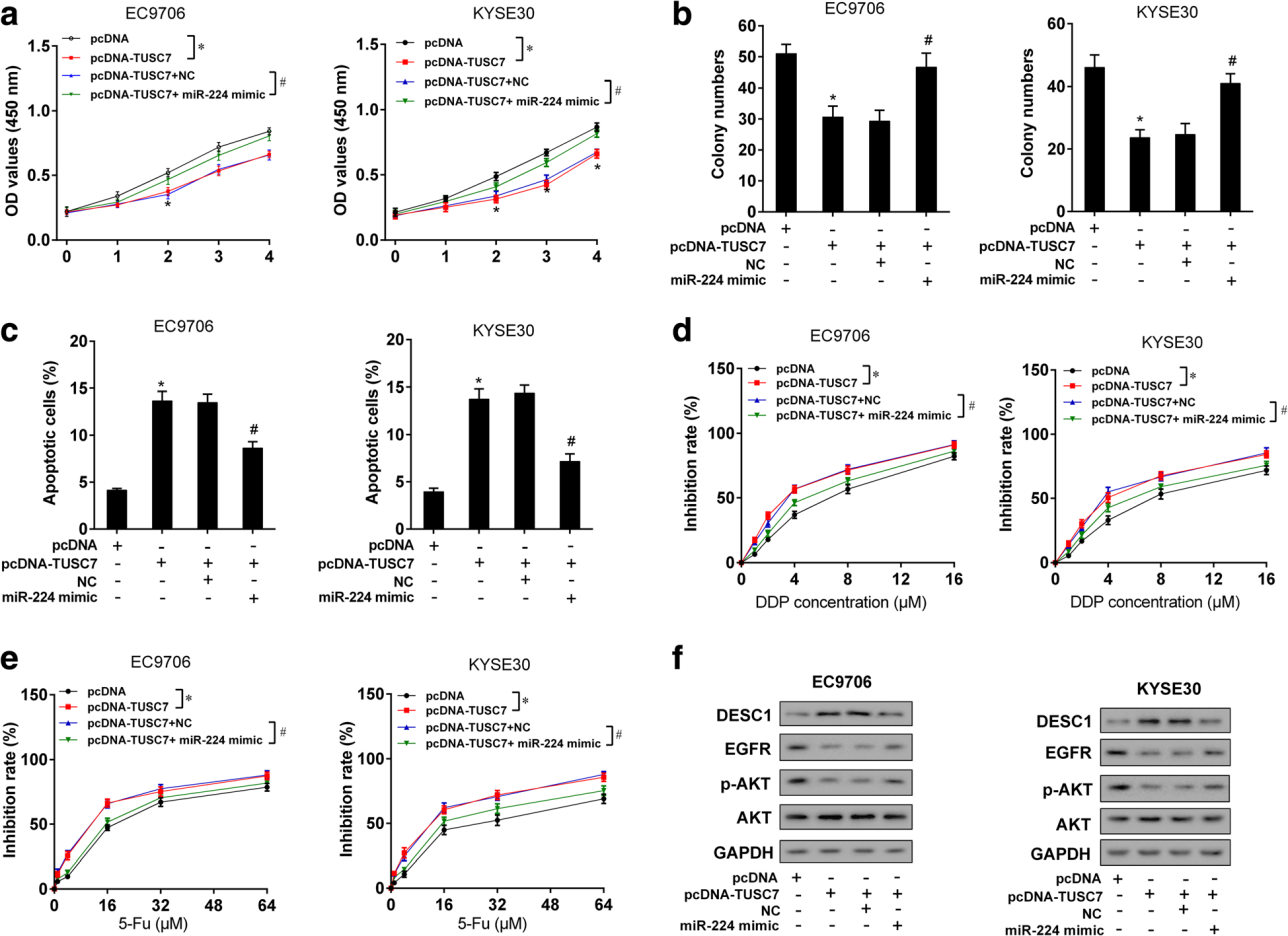
TUSC7 inhibited cell proliferation and chemotherapy resistance via miR-224/DESC1. EC9706 or KYSE30 cells were divided into four groups: pcDNA, pcDNA-TUSC7, pcDNA-TUSC7 + NC, and pcDNA-TUSC7 + miR-224 mimic groups. **a** MTT assay showed that miR-224 mimic reversed the inhibition effect of pcDNA-TUSC7 on cell proliferation. **b** Colony formation assay showed that miR-224 mimic reversed the inhibition effect of pcDNA-TUSC7 on colony formation. **c** Flow cytometry showed that miR-224 mimic reversed the promotion effect of pcDNA-TUSC7 on cell apoptosis. **d** After the treatment of cisplatin (0, 1, 2, 4, 8, 16 μM) for 48 h, miR-224 mimic reversed the inhibition effect of pcDNA-TUSC7 on chemotherapy resistance. **e** After the treatment of 5-Fu (0, 1, 4, 16, 32, 64 μM) for 48 h, miR-224 mimic reversed the inhibition effect of pcDNA-TUSC7 on chemotherapy resistance. **f** pcDNA-TUSC7 increased protein level of DESC1 and decreased the expression of EGFR and p-AKT, while miR-224 mimic reversed these effects

To figure out whether TUSC7 inhibited cell proliferation and chemotherapy resistance via miR-224/DESC1, EC9706 or KYSE30 cells were transfected with pcDNA, pcDNA-TUSC7, pcDNA-TUSC7 + NC, and pcDNA-TUSC7 + miR-224 mimic. We observed that miR-224 mimic reversed the inhibition effect of pcDNA-TUSC7 on cell proliferation (Fig. 6a), the inhibition effect of pcDNA-TUSC7 on colony formation (Fig. 6b), the promotion effect of pcDNA-TUSC7 on cell apoptosis (Fig. 6c), and the inhibition effect of pcDNA-TUSC7 on chemotherapy resistance (Fig. 6d and e). In addition, pcDNA-TUSC7 increased protein level of DESC1 and decreased the expression of EGFR and p-AKT, while miR-224 mimic reversed these effects (Fig. 6f).

### DESC1 inhibited chemotherapy resistance of ESCC cells via EGFR/AKT

**Fig. 7:**
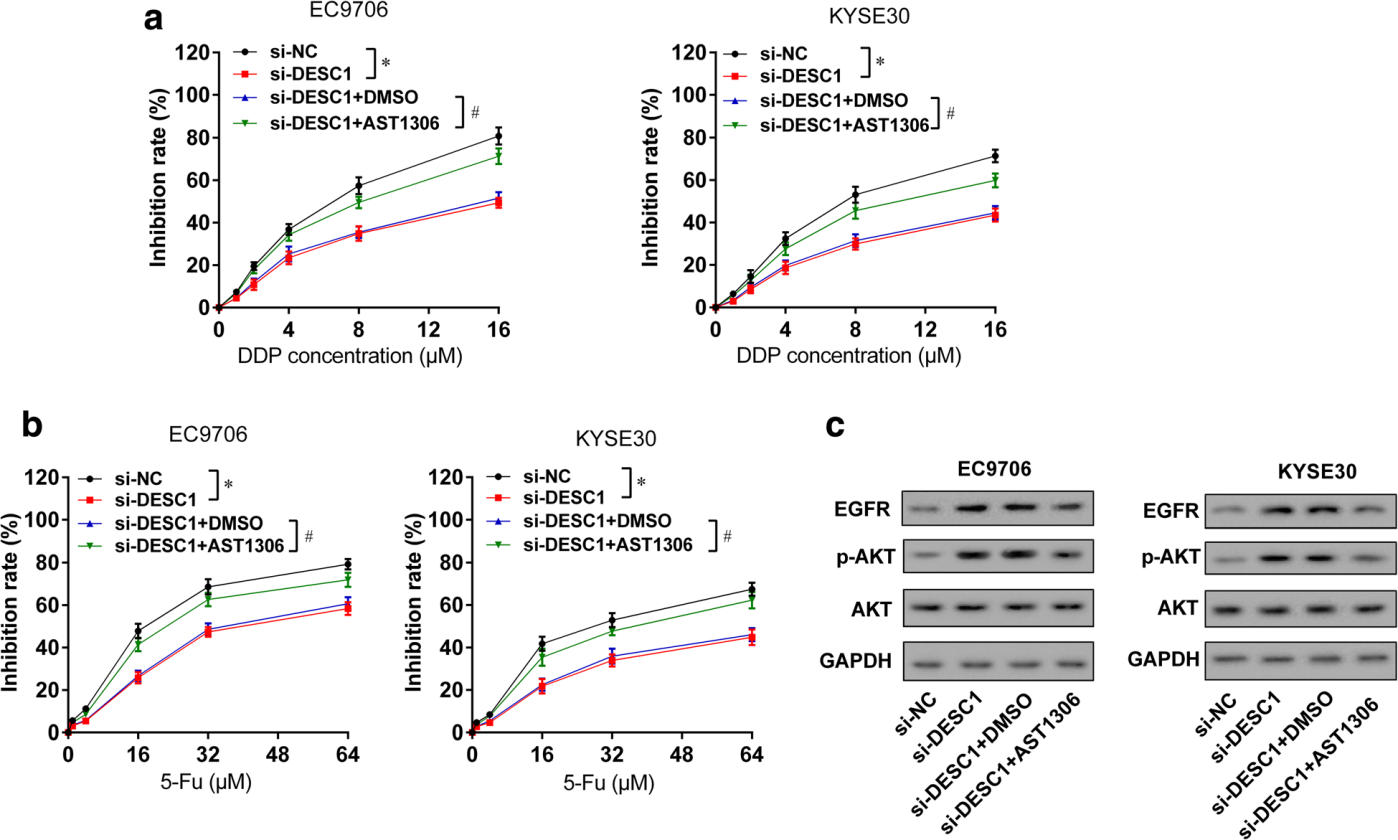
DESC1 inhibited chemotherapy resistance of ESCC cells via EGFR/AKT. EC9706 or KYSE30 cells were divided into four groups: si-NC, si-DESC1, si- DESC1 + DMSO, and si-DESC1 + AST1306 (EGFR inhibitor, 1 μM,24 h) groups. **a** After the treatment of cisplatin (0, 1, 2, 4, 8, 16 μM) for 48 h, si-DESC1 promoted chemotherapy resistance of ESCC cells, while AST1306 reversed this effect. **b** After the treatment of 5-Fu (0, 1, 4, 16, 32, 64 μM) for 48 h, si-DESC1 promoted chemotherapy resistance of ESCC cells, while AST1306 reversed this effect. **c** si-DESC1 upregulated the expressions of EGFR and p-AKT, while AST1306 reversed this effect

To figure out whether DESC1 inhibited chemotherapy resistance of ESCC cells via EGFR/AKT, EC9706 or KYSE30 cells were transfected with si-NC, si-DESC1, si- DESC1 + DMSO, and si-DESC1 + AST1306 (EGFR inhibitor) [24]. As shown in Fig. 7a and b, si-DESC1 promoted chemotherapy resistance of ESCC cells, while AST1306 reversed this effect. Also, si-DESC1 upregulated expressions of EGFR and p-AKT, while AST1306 reversed this effect (Fig. 7c). These results indicated that DESC1 inhibited chemotherapy resistance of ESCC cells via EGFR/AKT.

### TUSC7 regulated chemotherapy resistance of tumors in a xenograft model

**Fig. 8:**
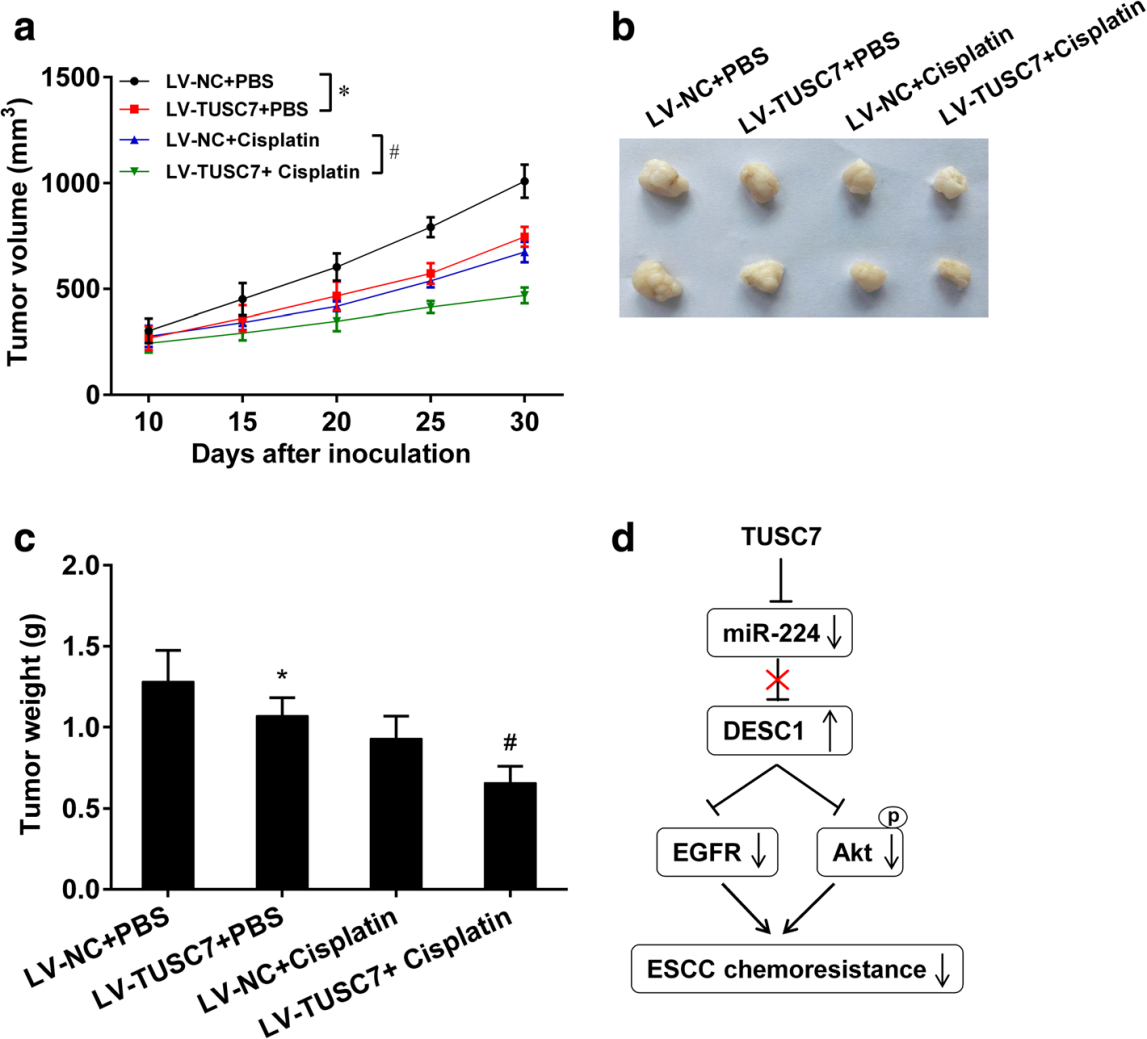
Observation of subcutaneous transplanted tumor of ESCC cells in nude mice. Five-week female BALB/c nude mice were divided into four groups with six mice in each group: LV-NC + PBS, LV-TUSC7 + PBS, LV-NC + cisplatin, and LV-TUSC7 + cisplatin groups. **a-c** Tumor volume, size and weight were measured. Overexpression of TUSC7 inhibited tumor growth and chemotherapy resistance. **d** Cascade diagram of signaling pathways

In order to confirm the regulatory role of TUSC7 in chemotherapy resistance in vivo, KYSE30 cells were transfected with LV-NC or LV-TUSC7 and subcutaneously injected into female nude mice. We observed that overexpression of TUSC7 inhibited tumor volume, size and weight (Fig. 8a–c). And overexpression of TUSC7 further decreased tumor volume and weight in cisplatin-treated mice (Fig. 8a and b). These results suggested that overexpression of TUSC7 inhibited tumor growth and chemotherapy resistance.

## Discussion

LncRNAs have been reported to be abnormally expressed in many cancers, and they have important roles in cancer and cancer-related diseases through different mechanisms [25, 26]. In order to identify whether lnc TUSC7 was abnormally expressed in ESCC and its effect on overall survival, qRT-PCR was used to measure the expression of TUSC7 in ESCC tissues and cells, and the Kaplan-Meier method was used to calculate overall survival. We further investigated the effect of TUSC7 on biological behavior of ESCC cells (EC9706 and KYSE30), and found overexpression of TUSC7/inhibition of miR-224 repressed proliferation of ESCC cells, promoted cell apoptosis, and inhibited chemotherapy resistance of ESCC cells by regulating DESC1. Moreover, we also observed overexpression of TUSC7 inhibited tumor volume and weight. These findings indicated that TUSC7 might act as a tumor suppressor and play an important role in the progression of ESCC.

Evidence has shown that lnc RNAs could exert biological function via competitively binding with miRNAs, and the analysis of bioinformatics softwares are commonly used to predict the combination sites between miRNAs and their target genes [20, 21]. According to the prediction of bioinformatics software in this study, there were combination sites between TUSC7 and miR-224. Dual-luciferase reporter gene assay and RIP assay were used to confirm the regulation of TUSC7 on miR-224 and proved TUSC7 could directly bind to miR-224. And overexpression of TUSC7 decreased miR-224 level, showing TUSC7 negatively regulated miR-224. We also proved miR-224 was upregulated in ESCC tissue, which was consistent with previous report [16]. At the same time, bioinformatics softwares also predicted that there were combination sites between miR-224 and DESC1. Dual-luciferase reporter gene assay and overexpression/inhibition of miR-224 proved miR-224 could directly bind to DESC1 and negatively modulate DESC1 level. And we found DESC1 level was downregulated in ESCC, which was consistent with previous report [11].

miR-224 played different roles in various cancers. It has been reported that miR-224 could act as an oncogenic miRNA in breast cancer [27], gastric cancer [28], non-small cell lung cancer [29], etc. Also, miR-224 could act as a tumor suppressor in prostate cancer [30], colorectal cancer [31], diffuse large B-cell lymphoma [32], etc. In 2015, He et al. first demonstrated that miR-224 was overexpressed in ESCC tissues and acted as an oncogenic miRNA in ESCC [16]. In this study, we found miR-224 was upregulated in ESCC tissues, and promoted proliferation of ESCC cells and reduced cell apoptosis via DESC1/EGFR/AKT pathway.

## Conclusions

In conclusion, our data proved that TUSC7 was downregulated and miR-224 was upregulated in ESCC, and high level of TUSC7 indicated better overall survival. In vitro experiments demonstrated that overexpression of TUSC7/inhibition of miR-224 repressed cell proliferation and chemotherapy resistance via DESC1/EGFR/AKT pathway, and in vivo experiments demonstrated that overexpression of TUSC7 decreased tumor growth and chemotherapy resistance.

## Abbreviations

CR: Complete response
DESC1: Differentially expressed in squamous cell carcinoma 1
EC: Esophageal cancer
ESCC: Esophageal squamous cell carcinoma
lncRNA: Long non-coding RNA
PD: Progressive disease
PR: Partial response
PVDF: Polyvinylidene difluoride
RECIST: Response Evaluation Criteria in Solid Tumors
*RIP*: *RNA immunoprecipitation*
SD: Stable disease
TTSP: Type II transmembrane serine protease
TUSC7: Tumor suppressor candidate 7
WT: Wild Type
